# Molecular Epidemiology of *Salmonella enterica* in Poultry in South Africa Using the Farm-to-Fork Approach

**DOI:** 10.1155/2022/5121273

**Published:** 2022-01-13

**Authors:** Melissa A. Ramtahal, Anou M. Somboro, Daniel G. Amoako, Akebe L. K. Abia, Keith Perrett, Linda A. Bester, Sabiha Y. Essack

**Affiliations:** ^1^Antimicrobial Research Unit, College of Health Sciences, University of KwaZulu-Natal, Durban 4000, South Africa; ^2^Biomedical Research Unit, School of Laboratory Medicine and Medical Sciences, College of Health Sciences, University of KwaZulu-Natal, Durban 4000, South Africa; ^3^Centre for Respiratory Diseases and Meningitis, National Institute for Communicable Diseases, Johannesburg 2131, South Africa; ^4^Epidemiology Section, KwaZulu-Natal Agriculture & Rural Development-Veterinary Service, Pietermaritzburg 3201, South Africa

## Abstract

The presence of the zoonotic pathogen *Salmonella* in the food supply chain poses a serious public health threat. This study describes the prevalence, susceptibility profiles, virulence patterns, and clonality of *Salmonella* from a poultry flock monitored over six weeks, using the farm-to-fork approach. *Salmonella* was isolated using selective media and confirmed to the genus and species level by real-time polymerase chain reaction (RT-PCR) of the *invA* and *iroB* genes, respectively. Antimicrobial susceptibility profiles were determined using Vitek-2 and the Kirby–Bauer disk diffusion method against a panel of 21 antibiotics recommended by the World Health Organisation Advisory Group on Integrated Surveillance of Antimicrobial Resistance (WHO-AGISAR). Selected virulence genes were identified by conventional PCR, and clonality was determined using enterobacterial repetitive intergenic consensus PCR (ERIC-PCR). *Salmonella* was present in 32.1% of the samples: on the farm (30.9%), at the abattoir (0.6%), and during house decontamination (0.6%). A total of 210 isolates contained the *invA* and *iroB* genes. Litter, faeces, and carcass rinsate isolates were classified as resistant to cefuroxime (45.2%), cefoxitin (1.9%), chloramphenicol (1.9%), nitrofurantoin (0.4%), pefloxacin (11.4%), and azithromycin (11%). Multidrug resistance (MDR) was observed among 3.8% of the isolates. All wastewater and 72.4% of carcass rinsate isolates were fully susceptible. All isolates harboured the *misL, orfL, pipD, stn, spiC, hilA*, and *sopB* virulence genes, while *pefA*, *spvA*, *spvB*, and *spvC* were absent. In addition, *fliC* was only present among the wastewater isolates. Various ERIC-PCR patterns were observed throughout the continuum with different subtypes, indicating the unrelated spread of *Salmonella*. This study concluded that poultry and the poultry environment serve as reservoirs for resistant and pathogenic *Salmonella*. However, there was no evidence of transmission along the farm-to-fork continuum.

## 1. Introduction

*Salmonella* infections remain one of the most common foodborne diseases, which constitutes a global public health concern [1]. As early as the 1950s, *Salmonella* was highlighted as an important zoonotic pathogen with economic implications by the World Health Organisation (WHO) and the Food and Agriculture Organisation (FAO) of the United Nations [2]. The genus *Salmonella* is divided into two species, *i.e., S. enterica* and *S. bongori*. *S. enterica* is also subdivided into six subspecies and comprises more than 2600 serovars [3]. The pathogenesis of *Salmonella* is mediated by various genes that promote host cell invasion, intracellular survival, and colonisation [4, 5].

*Salmonella* has been reported in intensive animal husbandry such as poultry production [6]. In poultry farms, flocks can become infected via vertical or horizontal transmission [7, 8]. Vertical transmission occurs when the reproductive tissues and organs of laying hens are infected with *Salmonella,* which is transmitted via the eggs to the progeny of the flock [3]. During horizontal transmission, poultry are exposed to contaminated litter, faeces, feed, water, equipment, other chickens, rodents, other animals, and farm personnel colonised/infected with *Salmonella* [9]. Food-producing animals are considered the primary sources of *Salmonella,* and their intestinal tract is the main reservoir for nontyphoidal *Salmonella* (NTS) strains [7]. Poultry products such as poultry meat, eggs and other poultry-derived products are common sources of NTS [1]. The sources and transmission routes of NTS in food are not well understood in low- and -middle-income countries (LMICs) due to the lack of integrated epidemiological surveillance systems [10, 11].

The extensive use of antimicrobials in poultry production and the emergence of MDR *Salmonella* strains, which can spread to humans via the food pathway, are a public health concern worldwide [7, 12]. In South Africa (SA), the use of antibiotics in commercial poultry farms has been associated with the development of MDR *Salmonella* strains in poultry intended for human consumption [2, 13]. *Salmonella* strains circulating among poultry have not been comprehensively investigated from the farm-to-fork approach [14, 15]. This study describes the prevalence, antimicrobial susceptibility profiles, virulence profiles, and clonality of *Salmonella* isolated along the farm-to-fork continuum to inform mitigation strategies.

## 2. Materials and Methods

### 2.1. Ethical Considerations

This study was part of an overarching research project on Antibiotic Resistance and One Health; ethical clearance was obtained from the Animal Research Ethics Committee (reference AREC 073/016PD) and the Biomedical Research Ethics Committee (reference BCA444/16) of the University of KwaZulu-Natal (UKZN). The study was also registered with the South African National Department of Agriculture, Forestry, and Fisheries (reference 12/11/1/5 (879)).

### 2.2. Study Population and Sample Strategy

#### 2.2.1. Study Area

An intensive poultry farm located in the uMgungundlovu district in Northern KwaZulu-Natal was identified and agreed to participate in this study. The sampling strategy adopted was according to the WHO-AGISAR guidelines [16].

#### 2.2.2. Sample Collection

A flock of one-day-old Cobb breed hatchlings, placed in a clean chicken house, was the target population. A total of 162 samples were collected using block sampling to ensure that the entire flock was represented within the poultry house over a six-week period. The sample collection included the following: weekly poultry faeces and litter, truck, and crate swabs when chickens were transported to the abattoir; carcass rinsate during slaughter at the abattoir; ceca from postslaughter and ready-to-cook packaged samples of the whole carcass, neck, and thigh. The wastewater sample was collected from the main drainpipe during house cleaning after the flock was removed. Briefly, ten litter and ten faecal samples (5 g of each sample) were collected per week (5 weeks = 50 litter, 50 faeces). On week 5, ten samples of each of the following were collected: truck swabs, crate swabs, ceca (25 g of each ceca sample), neck (25 g of each neck sample), thighs (25 g of each thigh sample), and whole carcasses. A single 40 ml sample was collected from carcass rinsate during slaughter and wastewater during the house decontamination. All samples were collected aseptically into sterile containers, labelled clearly, placed into cooler boxes, then transported immediately to the UKZN Antimicrobial Research Unit (ARU) laboratory. Samples were processed within four to six hours of collection. *Salmonella* prevalence was calculated as a percentage of *Salmonella* culture-positive samples among the total number of samples collected.

#### 2.2.3. Sample Processing and Isolation of *Salmonella*

The samples were inoculated in 40 ml of nonselective tryptic soy broth (TSB) (Oxoid Ltd., Basingstoke ﻿Hampshire, UK) and incubated for two hours at 37°C with shaking at 100 rpm. Thereafter, 0.1 ml was inoculated into 9.9 ml of Rappaport-Vassiliadis broth (Oxoid Ltd., Basingstoke, UK), a selective enrichment media for *Salmonella* and incubated at 42°C for 24 hours [17]. A loopful of the sample was streaked onto Brilliance *Salmonella* Agar (Oxoid Ltd., Basingstoke ﻿Hampshire, England) and incubated at 37°C for 24 hours [18]. Single presumptive purple/pink colonies typical of *Salmonella* were picked and subsequently streaked onto Hektoen Enteric Agar (HEA) (Oxoid Ltd., Basingstoke ﻿Hampshire, England) and *Salmonella* Shigella agar (SSA) (Oxoid Ltd., Basingstoke ﻿Hampshire, England) plates and incubated at 37°C for 24 hours. Culture plates were examined for the presence of typical colonies based on morphological characteristics, i.e., clear colonies with a black centre on HEA and SSA. Single colonies were subcultured onto nutrient agar and subsequently stored in TSB supplemented with 10% glycerol (Merck, USA) at −60°C until further analysis. *Salmonella enterica* subsp. *enterica* serovar Choleraesuis ATCC 10708 (ATCC, Manassas, Virginia, USA) was included as a control strain.

### 2.3. Isolation and Identification of *Salmonella*

#### 2.3.1. Phenotypic Confirmation of *Salmonella*

All presumptive stored *Salmonella* colonies were recovered from the freezer; subcultured and pure colonies on nutrient agar were subjected to biochemical identification by the catalase test using 3% H_2_O_2_ and the oxidase test using test strips (Sigma-Aldrich, St. Louis, USA). Further biochemical confirmation was performed using the API 20E kit (bioMérieux, Marcy I'Etoile, France) according to the manufacturer's instructions. *Salmonella enterica* subsp. *enterica* serovar Choleraesuis ATCC 10708 (ATCC, Manassas, Virginia, USA) was used as a control strain. All isolates that were phenotypically confirmed as *Salmonella* species (spp.) were subjected to further genotypic confirmation.

#### 2.3.2. Molecular Confirmation of *Salmonella*

DNA was extracted using the boiling method [19]. Colonies from an overnight culture grown on nutrient agar were briefly suspended into 200 *μ*l of distilled water and lysed at 100°C for 15 minutes. This was followed by centrifugation at 13000 rpm for 5 minutes; thereafter, 120 *μ*l of supernatant was transferred to a clean microcentrifuge tube and stored at 4°C until further analysis.

Isolates were genotypically confirmed as *Salmonella* using RT-PCR of the genus-specific *invA* gene (284 bp) and the *S. enterica* species-specific *iroB* gene (606 bp), using the QuantStudio 5 RT-PCR machine (Thermo Fisher Scientific, Waltham, MA, USA). The following primer sequences were used for *invA* F: 5′ GTGAAATTATCGCCACGTTCGGGCAA 3′and *invA* R: 5′ TCATCGCACCGTCAAAGGAACC 3′; *iroB* F: 5′ TGCGTATTCTGTTTGTCGGTCC 3′and *iroB* R: 5′ TACGTTCCCACCATTCTTCCC 3' [20, 21]. A 10 ul reaction mixture containing 5 *μ*l of Luna® Universal qPCR Master Mix (New England Biolabs, Ipswich, MA, USA), 0.5 *μ*l of each of the forward and reverse primers (20 *μ*M) (Inqaba Biotechnical Industries (Pty) Ltd., Pretoria, South Africa), 1 *μ*l of nuclease-free water (Lonza Rockland, ME, USA), and 3 *μ*l of template DNA was prepared. The following cycling conditions were used: an initial activation at 95°C for 10 minutes, followed by 50 cycles of denaturation at 95°C for 10 seconds (s), annealing at 64°C (15 s), extension at 72°C (25 s), and a final extension at 72°C for 5 min. Melting was done by ramping from 72 to 90°C, with a 0.1°C rise at each step, a premelt hold for 90 s in the first step, followed by a hold for 2 s in the next steps [22]. Real-time melt curves were obtained and the presence of the respective genes was analysed. The melting temperatures for the genes ranged from 85 to 86°C for *invA* and 88 to 89°C for *iroB*. *Salmonella enterica* subsp. *enterica* serovar Choleraesuis ATCC 10708 (ATCC, Manassas, Virginia, USA) was used as a positive control reference strain and a tube containing nuclease-free water (Thermo Fisher Scientific, Vilnius, ﻿Lithuania) instead of template DNA was included as a negative control.

### 2.4. Antimicrobial Susceptibility Testing (AST)

AST was carried out using the automated Vitek-2 system according to the manufacturer's instructions and the Kirby–Bauer disk diffusion method (for antibiotics absent from the Vitek-2 panel) following the established Clinical and Laboratory Standards Institute (CLSI) guidelines [23]. A panel of 21 antibiotics (Oxoid Ltd., Basingstoke Hampshire, UK) was tested following the WHO-AGISAR recommendations, as follows: ampicillin (10 *μ*g), amoxicillin (10 *μ*g), amoxicillin/clavulanate (10/20 *μ*g), cefuroxime (30 *μ*g), cefoxitin (30 *μ*g), cefotaxime (30 *μ*g), ceftazidime (30 *μ*g), cefepime (30 *μ*g), imipenem (10 *μ*g), meropenem (10 *μ*g), gentamicin (10 *μ*g), amikacin (30 *μ*g), chloramphenicol (30 *μ*g), azithromycin (15 *μ*g), nitrofurantoin (300 *μ*g), nalidixic acid (30 *μ*g), ciprofloxacin (5 *μ*g), pefloxacin (5 *μ*g), tetracycline (30 *μ*g), tigecycline (15 *μ*g), and trimethoprim/sulfamethoxazole (1.25/23.75 *µ*g). The WHO-AGISAR panel of antibiotics recommended for testing is based on well-established monitoring and surveillance systems on antimicrobial resistance in foodborne bacteria. The selection of antibiotics provides a harmonised standard that allows for data comparison between laboratories and countries [16]. In brief, a bacterial inoculum matched to a 0.5 McFarland standard was prepared from a pure overnight culture. For Vitek-2, the inoculum and AST-N255 cartridges were loaded into the instrument and programmed to run overnight. For disk diffusion, the suspension was evenly spread on a Mueller–Hinton agar plate using a sterile cotton swab and allowed to air-dry before dispensing the antibiotic disks onto the surface. Plates were incubated at 37°C for 18–24 hours. The minimum inhibitory concentrations (MIC) were determined by Vitek-2 and inhibition zones were measured for disk diffusion. The results were interpreted as susceptible, intermediate, or resistant according to the CLSI breakpoints [23]. *E. coli* ATCC 25922 (ATCC, Manassas, Virginia, USA) was used as a quality control strain. Isolates were classified as MDR if they displayed resistance to one or more antibiotics belonging to three or more different antibiotic classes [24, 25].

### 2.5. Molecular Detection of Virulence Factors

#### 2.5.1. DNA Extraction

Genomic DNA was purified from bacterial cultures grown overnight in 1 ml of TSB. The genomic DNA GeneJET extraction kit (Thermo Fisher Scientific, Vilnius, ﻿Lithuania) was used, following the manufacturer's instructions for Gram-negative bacteria. All DNA was stored at −20°C until further analysis by conventional PCR for virulence genes and clonality by ERIC-PCR.

#### 2.5.2. Detection of Virulence Genes

Twelve virulence genes were analysed by conventional PCR using specific primer pairs as described previously [4, 26, 27]. The primer sequences, cycling conditions, and product sizes are listed in Table 1. All PCR reactions were performed in a final volume of 15 *μ*l containing the following: 8 *μ*l of DreamTaq Master Mix (Thermo Fisher Scientific, Vilnius, ﻿Lithuania), 0.5 *μ*l of each the forward and reverse primers (20 *μ*M), 3.5 *μ*l of nuclease-free water (Thermo Fisher Scientific, Vilnius, ﻿Lithuania), and 2.5 *μ*l of the template DNA. *Salmonella enterica* subsp. *enterica* serovar Choleraesuis ATCC 10708 was used as quality control. The amplified PCR products were electrophoresed on a 1.5% agarose (Lonza Rockland, ME, USA) gel, containing 5 *μ*l of ethidium bromide (Sigma-Aldrich, St. Louis, MO, USA) in 1X TAE buffer (BioConcept Ltd., Allschwil, Switzerland), at 100 volts (V) for 1 hour. The bands were visualised using the Bio-Rad gel documentation system (Bio-Rad, Hercules, CA, USA) and Image software (Bio-Rad, Hercules, CA, USA). A 100 bp molecular weight ladder (New England Biolabs, Ipswich, MA, USA) was used to determine the size of the PCR fragments. *Salmonella enterica* subsp. *enterica* serovar Choleraesuis ATCC 10708 (ATCC, Manassas, Virginia, USA) was used as a positive control strain and a tube containing nuclease-free water (Thermo Fisher Scientific, Vilnius, ﻿Lithuania), instead of template DNA, was included as a negative control.

### 2.6. Determination of the Clonal Relationship Using Enterobacterial Repetitive Intergenic Consensus (ERIC-PCR)

For ERIC-PCR, universal primers ERIC-1R (5' -ATGTAAGCTCCTGGGGATTCAC- 3′) and ERIC-2 (5' -AAGTAAGTGACTGGGGTGAGCG- 3′) (Inqaba Biotechnical Industries (Pty) Ltd., Pretoria, South Africa) were used. The 25 *μ*l reaction mixture consisted of 12.5 *μ*l DreamTaq master Mix (Thermo Fisher Scientific, Vilnius, ﻿Lithuania), 0.1 *μ*l ERIC-R1 primer (100 *μ*M), 0.1 *μ*l ERIC-2 primer (100 *μ*M), 9.3 *μ*l nuclease-free water (Thermo Fisher Scientific, Vilnius, ﻿Lithuania), and 3 *μ*l template DNA. The following cycling conditions were used: initial denaturation at 95°C for 2 minutes followed by 34 cycles of denaturation at 90°C for 30 s, annealing at 52°C for 1 minute, extension at 65°C for 8 minutes, and a final extension at 65°C for 16 minutes [30]. *Salmonella enterica* subsp. *enterica* serovar Choleraesuis ATCC 10708 (ATCC, Manassas, Virginia, USA) was included as a quality control strain. An aliquot of the amplified products was separated on a 1.5% agarose gel, containing 5 *μ*l of ethidium bromide (Sigma-Aldrich, St. Louis, MO, USA) in 1X TAE buffer (BioConcept Ltd., Allschwil, Switzerland), set at 75 V for 3 hours. The bands were visualised using the Bio-Rad gel documentation system (Bio-Rad, Hercules, CA, USA) and image software (Bio-Rad, Hercules, CA, USA). Moreover, 100 bp and 1 Kb molecular weight ladders (New England Biolabs, Ipswich, MA, USA) were included on each gel. BioNumerics software version 6.6 (Applied Maths, Sint-Martens-Latem, Belgium) was used to construct a dendrogram using the unweighted pair group method with arithmetic mean (UPGMA) and Dice's coefficient set at 0.5% optimisation and 1% tolerance. Isolates were grouped based on a similarity index of 70% [31].

## 3. Results

### 3.1. *Salmonella* Prevalence

*Salmonella* was isolated from 32.1% (52/162) of the samples collected along the farm-to-fork continuum (Table 2). *Salmonella* was isolated as follows: on the farm: week 2, litter (10/10) and faeces (10/10); week 3, litter (10/10); week 5, litter (10/10) and faeces (10/10); at the abattoir during slaughter: carcass rinsate (1/1); week 6 during house decontamination, wastewater (1/1). *Salmonella* was not isolated from the following samples: week 1: litter (0/10) and faeces (0/10); week 3, faeces (0/10); week 4, litter (0/10) and faeces (0/10); week 5: trucks (0/10), crates (0/10), and ceca (0/10) and retail: neck (0/10), thigh (0/10), and whole carcass (0/10). The overall prevalence of *Salmonella* on the farm was 30.9% (50/162), consisting of 18.5% for litter (30/162) and 12.4% faeces (20/162). *Salmonella* also isolated from carcass rinsate was 0.6% (1/162) and wastewater 0.6% (1/162) (Table 2). A total of 210 isolates (Table S1) were confirmed as *Salmonella enterica* spp. by biochemical tests and using RT-PCR.

### 3.2. Antimicrobial Susceptibility Tests (ASTs)

The antimicrobial susceptibility profiles of the *Salmonella* isolates are shown in Table 3. Of the total isolates, 51% (107/210) were resistant to at least one antibiotic and of these, 3.8% (8/210) were categorised as MDR (Table 4). MDR isolates exhibited resistance among 2–4 different antibiotic classes (Tables 3 and 4). Of the total isolates, 24.3% (51/210) were classified as intermediate and 24.8% (52/210) were susceptible to all antibiotics (Table 3).

**Figure 1:**
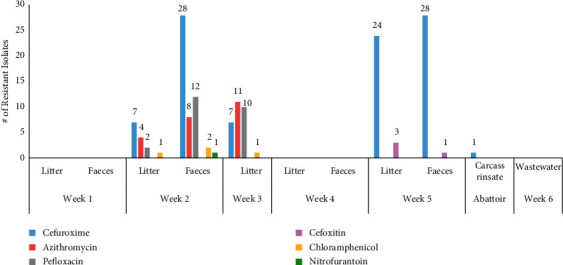
Distribution of resistant isolates stratified by source over the study period: on the farm, weeks 1–5 isolates consisted of litter and faeces; at the abattoir, carcass rinsate isolates were present.

The isolates were resistant to the following antibiotics: cefuroxime (45.2%, 95/210), cefoxitin (1.9%, 4/210), chloramphenicol (1.9%, 4/210), nitrofurantoin (0.4%, 1/210), pefloxacin (11.4%, 24/210), and azithromycin (11%, 23/210). The isolates were also classified as intermediate to the following antibiotics: 30% (63/210) to cefuroxime, 71.9% (151/210) to cefoxitin, 18.6% (39/210) to nitrofurantoin, 1.4% (3/210) to amoxicillin, 18.6% (39/210) to chloramphenicol, and 4.8% (10/210) to nalidixic acid (Table 5). The distribution of resistant isolates obtained from different sources is shown in Figure 1. All isolates were susceptible to tetracycline, cefotaxime, ceftazidime, cefepime, gentamicin, ampicillin, amikacin, meropenem, imipenem, tigecycline, ciprofloxacin, trimethoprim/sulfamethoxazole, and amoxicillin/clavulanic acid (Table 5).

### 3.3. Virulence Genes

Eight of the 12 virulence genes were detected among the isolates. The *misL, orfL, pipD, stn, spiC, hilA,* and *sopB* genes were present in all isolates from all sources, whereas *pefA*, *spvA*, *spvB,* and *spvC* were absent. The chromosomal virulence gene *fliC* was only present in the week 6 wastewater isolates.

### 3.4. ERIC-PCR

The ERIC-PCR yielded patterns consisting of 3–13 bands, with sizes ranging from 150 bp to 3 Kb. Diverse patterns were observed along the continuum. Clusters were source-specific based on a similarity index of 70% (Figure S1).

## 4. Discussion

The contamination of poultry and poultry products with *Salmonella* can occur at various stages of the production process. Although there are food safety standards, *Salmonella* continues to persist in the poultry processing industry [32].

### 4.1. Prevalence

In this study, an overall *Salmonella* prevalence of 32.1% was detected. The results are in agreement with those in previous studies conducted in broiler poultry farms in other countries that have reported a similar prevalence of *Salmonella* of 35% in Morocco and 31.25% in Bangladesh [33, 34]. The results of this study are also higher than those reported in the European Union (EU) (Finland and Sweden: <1%; Ireland: 19.8%), Asia (China: 12.6%; Korea: 15.3%), and South America (Columbia: 17.8%) [35–38]. Studies done in the USA and India along the farm-to-fork pathway reported an overall lower prevalence of 18.1% and 25.8%, respectively, than that in the current study [39, 40]. The proportion of *Salmonella* isolated from poultry can be influenced and varies by geographical location, environmental contamination, nature of farm management systems, type of production systems, breed of the flock, age of birds, sample size, sampling procedures, and the methods used for isolation [6, 11]. The biological nature of *Salmonella* in an infected host and its shedding pattern can also influence its isolation frequency, which may be seasonal and determined by environmental factors [25]. After contamination in poultry farms, *Salmonella* can colonise the intestines of chickens and result in contamination of the carcass during the slaughtering and meat processing stages [32].

In this study, *Salmonella* was not detected on the farm during week 1 and week 4. Sporadic isolation may be associated with various factors on the farm. *Salmonella* colonisation in chickens can be influenced by factors such as the age of the chicken, physiological and environmental stresses, survival of *Salmonella* through the gastric barrier, health and disease status of the chickens, vaccination, use of antimicrobials as growth promoters and prophylaxis, diet, and genetic background of the chickens [8, 41, 42]. In a previous study conducted in Ethiopia, young chicks were infected with 10^7−9^ CFU/ml of *S. typhimurium*. *Salmonella* was detected two weeks postinfection, suggesting that the immunity of young chicks can provide early protection. However, over time, at four weeks, a decline was observed, indicating that resistance to *Salmonella* infection by older chickens may be associated with the activation of cellular and humoral immunity [43]. This scenario may provide information on the immune status of the poultry flock evaluated in our study.

### 4.2. Antimicrobial Susceptibility

*Salmonella* strains found in poultry have also developed resistance to antibiotics, which are crucial for treating human infections [7]. In SA, antibiotics among livestock are used mainly in intensively farmed poultry and pigs. The AST results revealed that 51% (107/210) of the isolates were resistant to one or more antibiotics, and 3.8% (8/210) were classified as MDR. Resistance was detected for six of the 21 antibiotics tested, which belonged to classes of antibiotics (cephalosporins, macrolides, and quinolones) that are used in the SA poultry production. In comparison to our study, higher rates of MDR *Salmonella* among poultry isolates have previously been reported in Africa (12.1%–100%), Asia (34.72–43.1%), the USA (9.5–18%), and the EU (38.2%) [12, 33, 37, 44–48].

In this study, resistance to the second-generation cephalosporins, cefuroxime (45.2%) and cefoxitin (1.9%), among isolates that were susceptible to other *β*-lactam antibiotics such as ampicillin was observed. A similar resistance phenotype was reported in Saudi Arabia, where clinical and environmental samples showed resistance to cefuroxime (90.9%) and cefoxitin (87.9%), while most isolates were susceptible to *β*-lactams [49]. Previous studies in Africa, Australia, and the USA have reported a higher frequency of resistance to cefuroxime or cefoxitin among *Salmonella*-positive poultry isolates [14, 39, 46, 50].

In 2015, pefloxacin was recommended as a reliable surrogate marker to identify susceptibility to fluoroquinolones, as tests with nalidixic acid and ciprofloxacin disks do not reliably detect resistance at low levels in *Salmonella* spp. [51–55]. This was observed in this study where there was 100% susceptibility to ciprofloxacin and 4.8% (10/210) intermediate to nalidixic acid compared with 11.4% (24/210) resistance to pefloxacin (Table 5), indicating that antibiotic panels may impact the resistance profiles observed. A similar scenario was reported in India by Prabhurajan et al. [56], where a higher rate of resistance to pefloxacin than that of nalidixic acid was observed among clinical *Salmonella* isolates. It was concluded that different mechanisms and mutations associated with fluoroquinolone resistance can influence atypical resistance phenotypes [56]. A systematic review of fluoroquinolone resistance in *Salmonella* in Africa by Taddesse et al. [57] also reported that *Salmonella* strains susceptible to nalidixic acid and resistant to ciprofloxacin have been identified among NTS serovars. The review also indicated that although reports on the distribution of *Salmonella* strains with unusual AST phenotypes are scarce, these strains occur in Africa [57].

Previous studies have reported varying levels of resistance or susceptibility to nalidixic acid, ciprofloxacin, and pefloxacin among isolates, attributed to the varying resistance mechanisms of fluoroquinolones. Different AST methodologies or reagents have also been identified as possible causes of this variation. Antibiotic discs from different manufacturers have given varying results and the narrow range to classify isolates as resistant or susceptible may introduce subjectivity when reading plates. The CLSI M100 guidelines indicate that no single antibiotic test can accurately detect all types of resistance associated with fluoroquinolones [7, 54, 58–60]. Automated systems used in laboratories may also have challenges in implementing new lower breakpoints for ciprofloxacin, and therefore, low-level resistance in isolates may be undetected [61].

In comparison to the results of the current study, higher rates of fluoroquinolone resistance (ciprofloxacin and nalidixic) have been reported in poultry in the EU (64.7% and 61.5%), Brazil (86.5% and 89%), and China (25.7% and 46.7%) [37, 45, 60]. However, *Salmonella* isolated from broilers in Canada (3% and <1%) and in the USA (0% and <1%) have shown a low resistance frequency to fluoroquinolones, which can be attributed to the restricted use of fluoroquinolones in poultry [39, 60, 62, 63].

In this study, low resistance frequencies were also observed for chloramphenicol (1.9%), nitrofurantoin (0.4%), and azithromycin (11%). Similarly, low rates of chloramphenicol resistance were reported among poultry isolates in Brazil (0.6%) and the USA (0.3%) [39, 60]. The low chloramphenicol and nitrofurantoin resistance in this study can be attributed to the prohibited use of these antibiotics in food animals in SA [64]. In South Korea, a slightly higher but comparable azithromycin resistance (17.9%) was reported compared to the results in the present study. In contrast, in the EU, USA, and Brazil, azithromycin resistance in food-producing animals and food products was either not detected or detected at a very low frequency (<1%) [12, 48, 60, 65]. Azithromycin is used for the treatment of MDR Salmonellosis in humans. Resistance to azithromycin observed in the present study may be associated with the authorised use of other macrolides such as tylosin on poultry farms in SA compared to the EU where macrolides are banned [12, 64, 66].

However, based on the resistant results obtained in this study associated with second-generation cephalosporins, quinolones, and fluoroquinolones, further molecular analysis of the genes that confer resistance and the mechanisms involved would be needed to explain the unusual AST phenotypes observed. This study also identified the presence of MDR *Salmonella* strains specifically at the farm level (Table 2). Antibiotic resistance continues to increase in LMICs, due to the injudicious use of antimicrobials as growth promoters and feed additives to strengthen the intestinal microflora and for the control, prophylaxis, and treatment of infectious diseases in animal husbandry [41]. In this study, *Salmonella* isolates were resistant to antibiotic classes that are listed as critically and highly important antimicrobials for human medicine by the WHO and as antimicrobials of veterinary importance by the World Organisation for Animal Health (OIE) [67, 68]. In Europe and the USA, many of these antibiotic classes are restricted or prohibited for use in food animals [63, 66]. In some African countries, antibiotics are used in food animals without strict regulations [14, 46]. However, in SA, regulations and policies exist, although enforcement remains challenging [64, 69]. The resistant *Salmonella* strains in food animals and food products can be transmitted to humans through the food chain, and this poses a health risk to consumers by causing infections that are more severe and difficult to treat [60, 70].

### 4.3. Virulence

The virulence genes present in *Salmonella* can determine the pathogenicity among the strains and serotypes circulating in poultry and the environment [26, 71]. In SA, information on the prevalence of *Salmonella* virulence genes found in poultry is limited, and only a few studies have investigated virulence in chickens [4, 21]. Gene clusters known as *Salmonella* pathogenicity islands (SPIs) are regions located on chromosomes that are distributed in the *Salmonella* genome, which are associated with virulence and enable efficient bacterial colonisation in the host. Genes such as *hilA* are found in SPI-1 that encode proteins involved in cell invasion [27]. Other genes located in the following SPIs, the SPI-2 gene *spiC, the* SPI-3 gene *misL, and the* SPI-4 gene *orfL,* are responsible for replication within macrophages [21, 28]. Genes located in SPI-5, such as *pipD* and *sopB,* promote enteritis and macrophage invasion accordingly [21, 27, 28, 72].

In this study, all *Salmonella* isolates possessed these virulence genes, indicating that healthy chickens and their environment serve as carriers of pathogenic *Salmonella* strains, which can cause systemic infection and enteric infection in the host [28]. Previous studies conducted in SA, UK, Egypt, and China have also reported the presence of these virulence genes in poultry [21, 26, 28, 72].

Other genes present on the *Salmonella* chromosomes found in this study were *stn* and *fliC,* which play a role in enterotoxin production and encode phase-1 flagellin, respectively [26]. All *Salmonella* isolates contained the *stn* gene, which has also been reported in all poultry isolates from studies conducted in China, Benin, and India [72–74]. However, the *fliC* gene in our study was only detected in the wastewater isolates obtained during the house decontamination. The *fliC* gene encodes for the flagellin protein and has been used as a target gene to determine antigenic specificity and genetic diversity in *Salmonella*. Studies done in Egypt, Malaysia, and Saudi Arabia described that *fliC* was serovar-specific for *S.* typhimurium*, S.* Kentucky, *S.* Aberdeen, *S.* Bergen, and *S.* Kedougou [26, 27, 75]. Serovars were not determined in this study.

### 4.4. Clonality

In Columbia, Herrera-Sanchez et al. [76] reported similar ERIC banding patterns consisting of 2—13 bands that ranged from 200 to 4000 bp in size whilst contrasting ERIC results of band sizes 15—1500 bp and 190—1430 bp were observed by Fendri et al. [77] in France and Oliveria et al. [78] in Brazil, respectively. Among the 210 isolates, there were various patterns and clusters. Isolates obtained from litter and faeces over the collection period clustered, indicating clonal spread on the farm. However, the carcass rinsate and wastewater isolates formed separate clusters. *Salmonella* transmission along the farm-to-fork continuum was not evident as unrelated strains from different sources belonged to different clusters based on a 70% similarity index. Of note, although ERIC-PCR has a shorter turnaround time, it is less discriminatory. Therefore, further studies involving more resolute typing approaches, such as serotyping and whole-genome sequencing (WGS), are recommended to elucidate the population structure of the strains.

## 5. Conclusion

Our study showed sporadic *Salmonella* contamination along the farm-to-fork pathway and demonstrated that healthy chickens could serve as a reservoir of resistant and pathogenic *Salmonella* strains, which harbour virulence factors and exhibit resistance to various antibiotics. The presence of MDR *Salmonella* strains among litter and faeces in this study indicates that the imprudent use of antibiotics on poultry farms could contribute to the increasing development and spread of antibiotic resistance in food animals and animal food products. Therefore, the initial steps to reduce and control *Salmonella* contamination of poultry meat should be taken at the farm level. SA has developed and implemented an Antimicrobial Resistance National Strategy Framework for 2018–2024 with the strategic objective of promoting the appropriate use of antibiotics in humans and animals using regulations. However, interventions to address antimicrobial use and resistance in food animals need to be expedited.

## Figures and Tables

**Table 1:** Primers used for the amplification of *Salmonella* virulence genes.

*Gene*	Primer sequence	Initial denaturation	# of cycles	Cycling conditions			Final extension	Product size (bp)	References
				Denaturation	Annealing	Extension			
*hilA*	F: 5′ CGGAAGCTTATTTG CGCCATGCTGAGGTAG 3′	94°C, 5 m	30	94°C, 1 m	65°C, 1 m	72°C, 1 m	72°C, 10 m	854	[26]
	R: 5′ GCATGGATCCCCG CGGCGAGATTGTG 3′								
*spiC*	F: 5′ CCTGGATAATGAC TATTGAT 3′	94°C, 3 m	30	94°C, 1 m	55°C, 1 m	72°C, 1 m	72°C, 5 m	309	[21, 28]
	R: 5′ AGTTTATGGTG ATTGCGTAT 3′								
*misL*	F:5′ GTCGGCGAATGC CGCGAATA 3′	94°C, 3 m	30	94°C, 1 m	55°C, 1 m	72°C, 1 m	72°C, 5 m	550	[21, 28]
	R: 5′ GCGCTGTTAAC GCTAATAGT 3′								
*orfL*	F: 5′ GGAGTATCGAT AAAGATGTT 3′	94°C, 3 m	30	94°C, 1 m	55°C, 1 m	72°C, 1 m	72°C, 5 m	350	[21, 28]
	R: 5′ GCGCGTAACGTC AGAATCAA 3′								
*pipD*	F: 5′ CGGCGATTCATG ACTTTGAT 3′	94°C, 5 m	34	94°C, 25°s	56°C, 30°s	72°C, 50°s	72°C, 5 m	400	[21, 28]
	R: 5′ CGTTATCATTCG GATCGTAA 3′								
*sopB*	F: 5′ TCAGAAGRCGTC TAACCACTC 3′	94°C, 3 m	30	94°C, 1 m	55°C, 1 m	72°C, 1 m	72°C, 5 m	517	[27]
	R: 5′ TACCGTCCTCA TGCACACTC 3′								
*stn*	F: 5′TTGTGTCGCTATCA CTGGCAACC 3′	94°C, 5 m	25	94°C, 1 m	59°C, 1 m	72°C, 1 m	72°C, 10 m	617	[26]
	R: 5′ ATTCGTAACCCG CTCTCGTCC 3′								
*fliC*	F: 5′ CGGTGTTGCCCA GGTTGGTAAT 3′	94°C, 3 m	30	94°C, 1 m	55°C, 1 m	72°C, 1.5 m	72°C, 10 m	620	[26]
	R: 5′ ACTGGTAAAGAT GGCT 3′								
*pefA*	F: 5′ TGTTTCCGGGCT TGTGCT 3′	94°C, 5 m	25	94°C, 55°s	55°C, 55°s	72°C, 55°s	72°C, 10 m	700	[26]
	R: 5′ CAGGGCATTTGC TGATTCTTCC 3′								
*spvA*	F: 5′GTCAGACCCGT AAACAGT 3′	94°C, 5 m	30	94°C, 30°s	60°C, 30°s	72°C, 1 m	72°C, 5 m	604	[29]
	R: 5′ GCACGCAGAG TACCCGCA 3′								
*spvB*	F: 5′ ACGCCTCAGCG ATCCGCA 3′	94°C, 5 m	30	94°C, 30 s	60°C, 30°s	72°C, 1 m	72°C, 5 m	1063	[29]
	R: 5′ GTACAACATCT CCGAGTA 3′								
*spvC*	F: 5′ CGGAAATACCA TCAAATA 3′	94°C, 5 m	30	93°C, 1 m	42°C, 1 m	72°C, 2 m	72°C, 4°m	669	[26]
	R: 5′ CCCAAACCCAT ACTTACTCTG 3′								

**Table 2:** Prevalence of *Salmonella* along the farm-to-fork continuum.

Week	Production stage	Type of sample	# of samples	*# Salmonella*-positive	Prevalence (%)
1 to 5	Farm: growth period	Litter	50	30	18.5
		Faeces	50	20	12.4
5	Transport and handling	Truck swabs	10	0	
		Crate swabs	10	0	
5	Slaughter	Abattoir: carcass rinsate	40 ml = 1	1	0.6
	Postslaughter	Caeca	10	0	
	Retail meat	Neck	10	0	
		Thigh	10	0	
		Whole carcass	10	0	
6	House decontamination	Wastewater	40 ml = 1	1	0.6
**Total**			**162**	**52**	**32.1**

**#**: number.

**Table 3:** Number of isolates with varying susceptibility profiles stratified by source.

Susceptibility profile		Week 2		Week 3		Week 5		Week 6	Total
Resistant	Intermediate	Litter	Faeces	Litter	Litter	Faeces	Carcass rinsate	Wastewater	
CXM	FOX	3	7	3	16	17	1		47
CXM	FOX-CHL	1	2	1					4
CXM	FOX-NIT		1	1	5	9			16
CXM	FOX-NIT-CHL		1						1
CXM	FOX-NIT-NA					1			1
AZM	CXM-FOX-CHL			2					2
AZM	CXM-FOX-NIT-CHL	1							1
PEF	CXM-FOX-CHL			1					1
PEF	CXM-FOX-NIT-CHL			1					1
CXM-FOX	—				1	1			2
CXM-FOX	NIT				2				2
CXM-CHL	FOX		1						1
CXM-AZM	FOX-CHL	1	3						4
CXM-AZM	FOX-NIT-CHL			1					1
CXM-PEF	FOX-CHL		3						3
CXM-PEF	FOX-CCHL-AMX		1						1
CXM-PEF	FOX-CHL-NA		2						2
CXM-PEF	FOX-CHL-NA-AMX		1						1
CXM-NIT	FOX-CHL		1						1
PEF-AZM	CXM-FOX-CHL			4					4
PEF-AZM	CXM-FOX-CHL-NA			2					2
PEF-AZM	CXM-FOX-NIT-CHL			1					1
CXM-PEF-AZM	FOX-CHL		2						2
CXM-PEF-AZM	FOX-NIT-CHL	1	1						2
CXM-PEF-AZM	FOX-NIT-CHL-NA-AMX		1						1
CXM-CHL-PEF-AZM	FOX-NA	1	1	1					3
—	CXM						2		2
—	CXM-NIT				1				1
—	CXM-FOX	12	2	8	5	2	5		34
—	CXM-FOX-CHL			3					3
—	CXM-FOX-NIT	10							10
—	CXM-FOX-NIT-CHL			1					1
**Susceptible**						21	31	52	
**Total**		**30**	**30**	**30**	**30**	**30**	**29**	**31**	**210**

CXM: cefuroxime; FOX: cefoxitin; CHL: chloramphenicol; NIT: nitrofurantoin; AZM: azithromycin; PEF: pefloxacin.

**Table 4:** Single and multiple antibiotic resistance patterns of *Salmonella* isolates.

Number of antibiotics	Resistance pattern (#)	# of isolates (%)	MDR (%)
1	CXM (69), AZM (3), PEF (2)	74 (35.2%)	
2	CXM-FOX (4), CXM-CHL (1), CXM-AZM (5), CXM-PEF (7), CXM-NIT (1), PEF-AZM (7)	25 (11.9%)	
3	CXM-PEF-AZM	5 (2.4%)	5 (2.4%)
4	CXM-CHL-PEF-AZM	3 (1.4%)	3 (1.4%)
**Total**		107 (51%)	8 (3.8%)

#: number; CXM: cefuroxime; FOX : cefoxitin; CHL : chloramphenicol; NIT : nitrofurantoin; AZM : azithromycin; PEF : pefloxacin.

**Table 5:** Antimicrobial susceptibility results for *Salmonella* isolates from poultry.

Antimicrobial class	Antimicrobials	# of isolates	Susceptibility profile		
			S (%)	I (%)	R (%)
Aminoglycosides	AMK	210	210 (100%)	0	0
	GEN	210	210 (100%)	0	0
Carbapenems	MER	210	210 (100%)	0	0
	IMP	210	210 (100%)	0	0
Cephalosporins	FOX (II)	210	55 (26.2%)	151 (71.9%)	4 (1.9%)
	CXM (II)	210	52 (24.8%)	63 (30%)	95 (45.2%)
	CTX (III)	210	210 (100%)	0	0
	CFZ (III)	210	210 (100%)	0	0
	FEP (IV)	210	210 (100%)	0	0
Macrolides	AZM	210	187 (89%)	0 (0%)	23 (11%)
Nitrofurans	NIT	210	170 (81%)	39 (18.6%)	1 (0.4%)
Penicillins	AMX	210	207 (98.6%)	3 (1.4%)	0
	AMP	210	210 (100%)	0	0
Phenicols	CHL	210	167 (79.5%)	39 (18.6%)	4 (1.9%)
Quinolones	NA	210	200 (95.2%)	10 (4.8%)	0
	PEF	210	186 (88.6%)	0 (0%)	24 (11.4%)
	CIP	210	210 (100%)	0	0
Tetracyclines	TET	210	210 (100%)	0	0
	TGC	210	210 (100%)	0	0
Other	AMC	210	210 (100%)	0	0
	SXT	210	210 (100%)	0	0

S: susceptible; I: intermediate; R: resistant; AMC: amoxicillin/clavulanic acid; AMK: amikacin; AMP: ampicillin; AZM: azithromycin; CIP: ciprofloxacin; CXM: cefuroxime; FOX: cefoxitin; CHL: chloramphenicol; CTX: cefotaxime; CFZ: ceftazidime; FEP: cefepime; GEN: gentamicin; IMP: imipenem; MER: meropenem; NIT: nitrofurantoin; PEF: pefloxacin; TET: tetracycline; TGC: tigecycline; SXT: trimethoprim/sulfamethoxazole.

## Data Availability

The data used to support the findings of this study are included within the article.

## Supplementary Materials

Supplementary Materials Table S1: source of *Salmonella* isolates. Figure S1: dendrogram of ERIC-PCR patterns constructed of *Salmonella enterica* isolates recovered from the farm-to-fork continuum. *Salmonella enterica* subsp. *enterica* serovar Choleraesuis ATCC 10708 was used as the quality control strain. .
