# Supplementary material for: Molecular Epidemiology of Salmonella enterica in Poultry in South Africa Using the Farm-to-Fork Approach
Source: Int J Microbiol. 2022 Jan 13;2022:5121273. doi: 10.1155/2022/5121273 (PMC8776487; doi:10.1155/2022/5121273)
Supplement: Supplementary Materials — Table S1: source of Salmonella isolates. Figure S1: dendrogram of ERIC-PCR patterns constructed of Salmonella enterica isolates recovered from the farm-to-fork continuum. Salmonella enterica subsp. enterica serovar Choleraesuis ATCC 10708 was used as the quality control strain. . [file 5121273.f1.zip › Supplementary Table S1.docx]

| **Table S1: Source of** *Salmonella* **isolates used in this study** | | | | | |  |
| --- | --- | --- | --- | --- | --- | --- |
|  |  |  |  |  | |  |
| **Week** | **Production Stage** | **Point of Collection** | **# Presumptive colonies** | | **#** *Salmonella* **isolates** | |
|  |  |  |  | |  | |
| 1 to 5 | Growth period | Litter | 131 | | 90 | |
|  |  | Faeces | 84 | | 60 | |
|  |  |  |  | |  | |
| 5 | Transport & handling | Truck | 0 | |  | |
|  |  | Crate | 0 | |  | |
|  |  |  |  | |  | |
| 5 | Slaughter | Abattoir: carcass rinsate | 40 | | 29 | |
|  | Post slaughter | Caeca | 0 | |  | |
|  | Retail meat | Neck | 0 | |  | |
|  |  | Thigh | 0 | |  | |
|  |  | Whole carcass | 0 | |  | |
|  |  |  |  | |  | |
| 6 | House decontamination | Wastewater | 40 | | 31 | |
|  |  |  |  |  | |  |
|  | **TOTAL** |  | **295** | | **210** | |
|  |  |  |  | |  | |
