# Supplementary figures and images for: Molecular Epidemiology of Salmonella enterica in Poultry in South Africa Using the Farm-to-Fork Approach
Source: Int J Microbiol. 2022 Jan 13;2022:5121273. doi: 10.1155/2022/5121273 (PMC8776487; doi:10.1155/2022/5121273)

## ERIC PCR

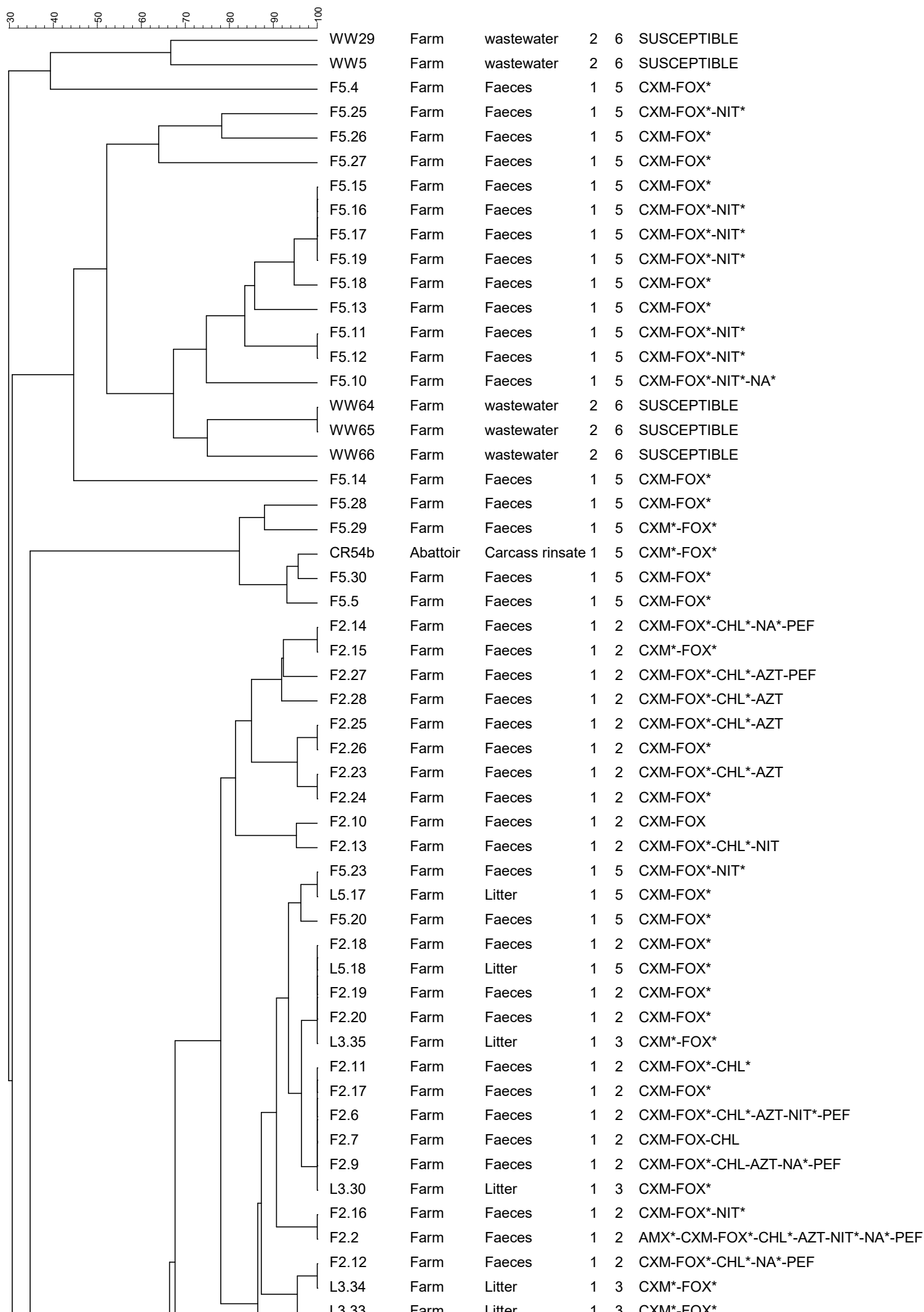

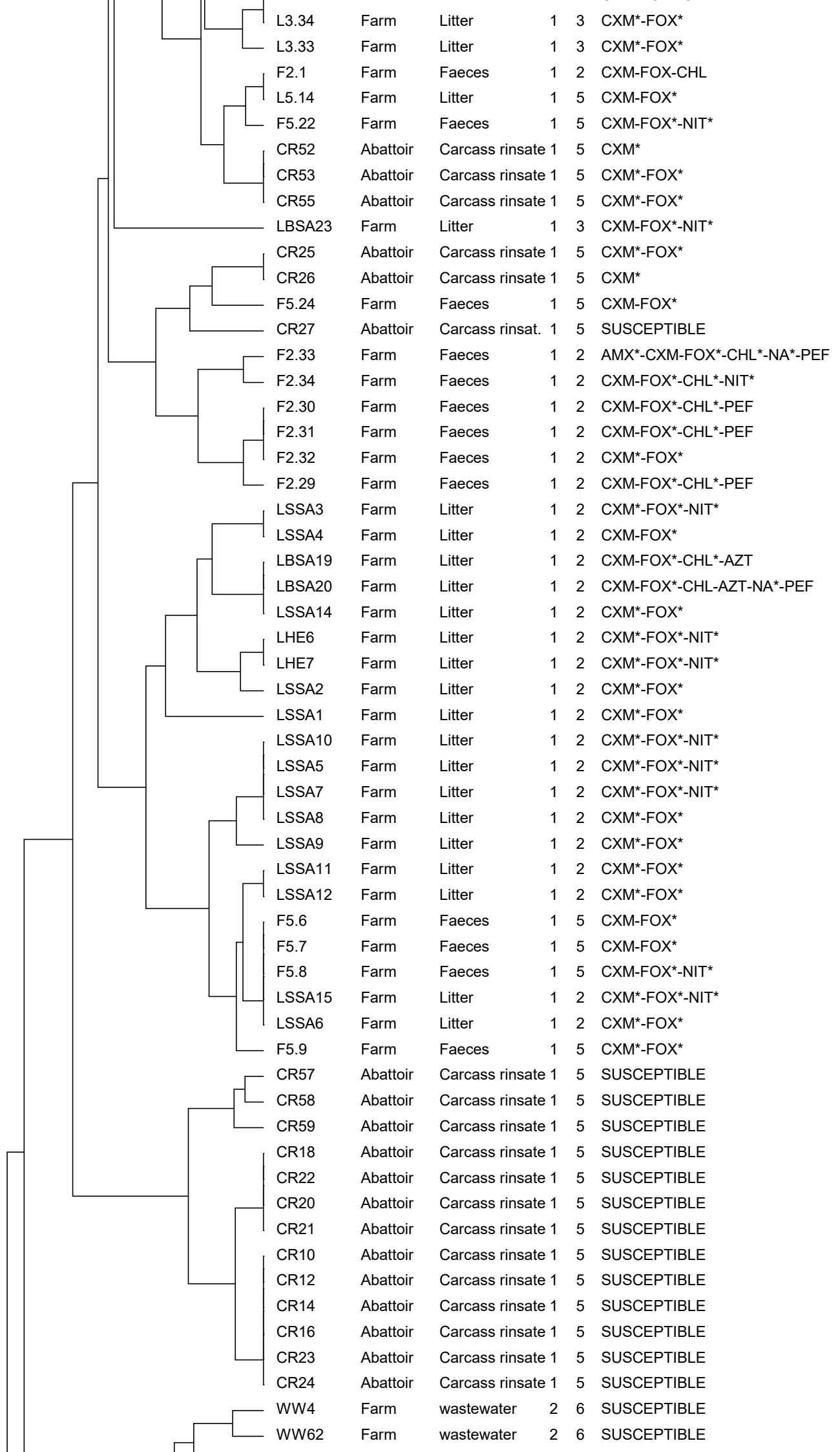

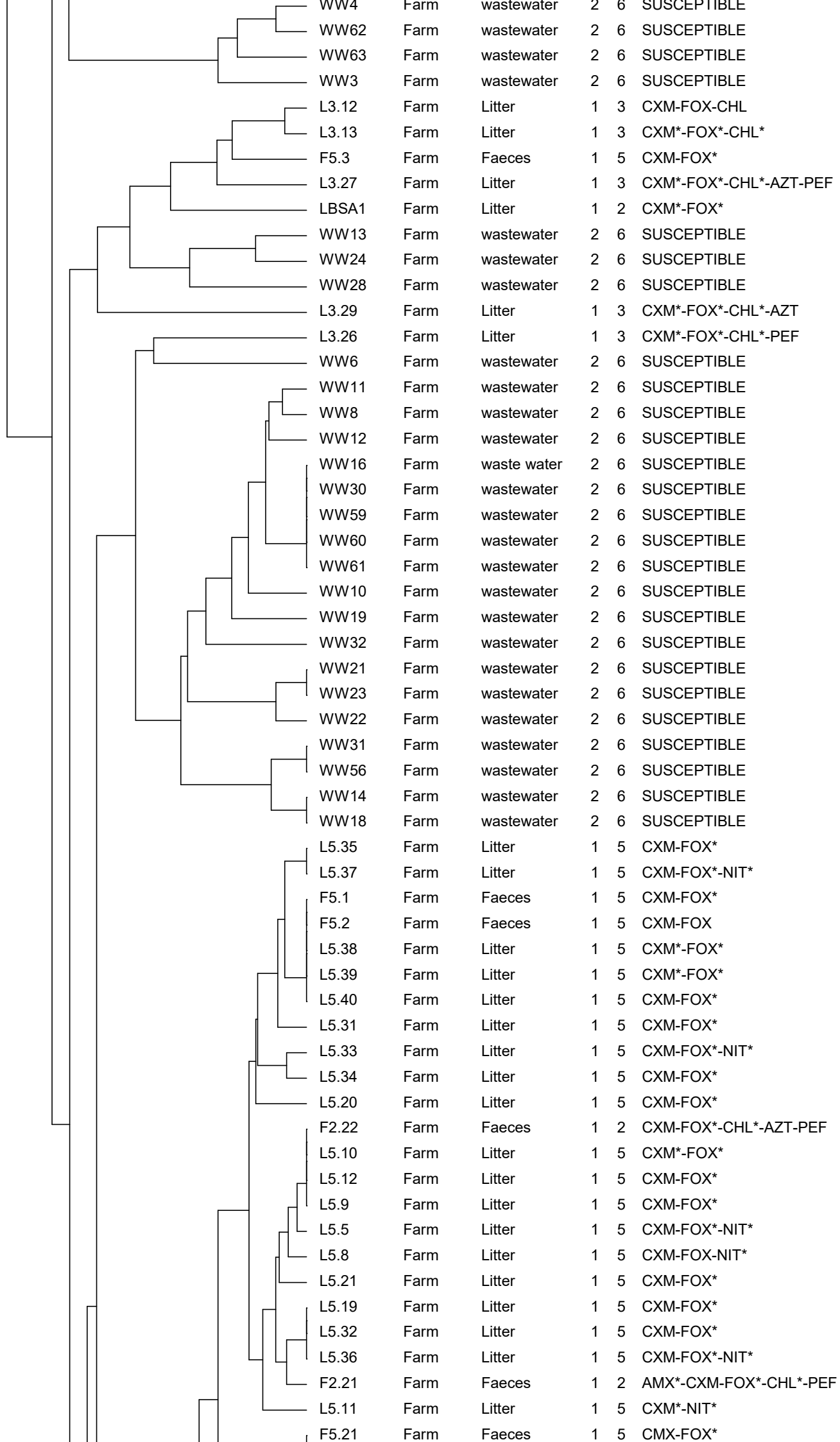

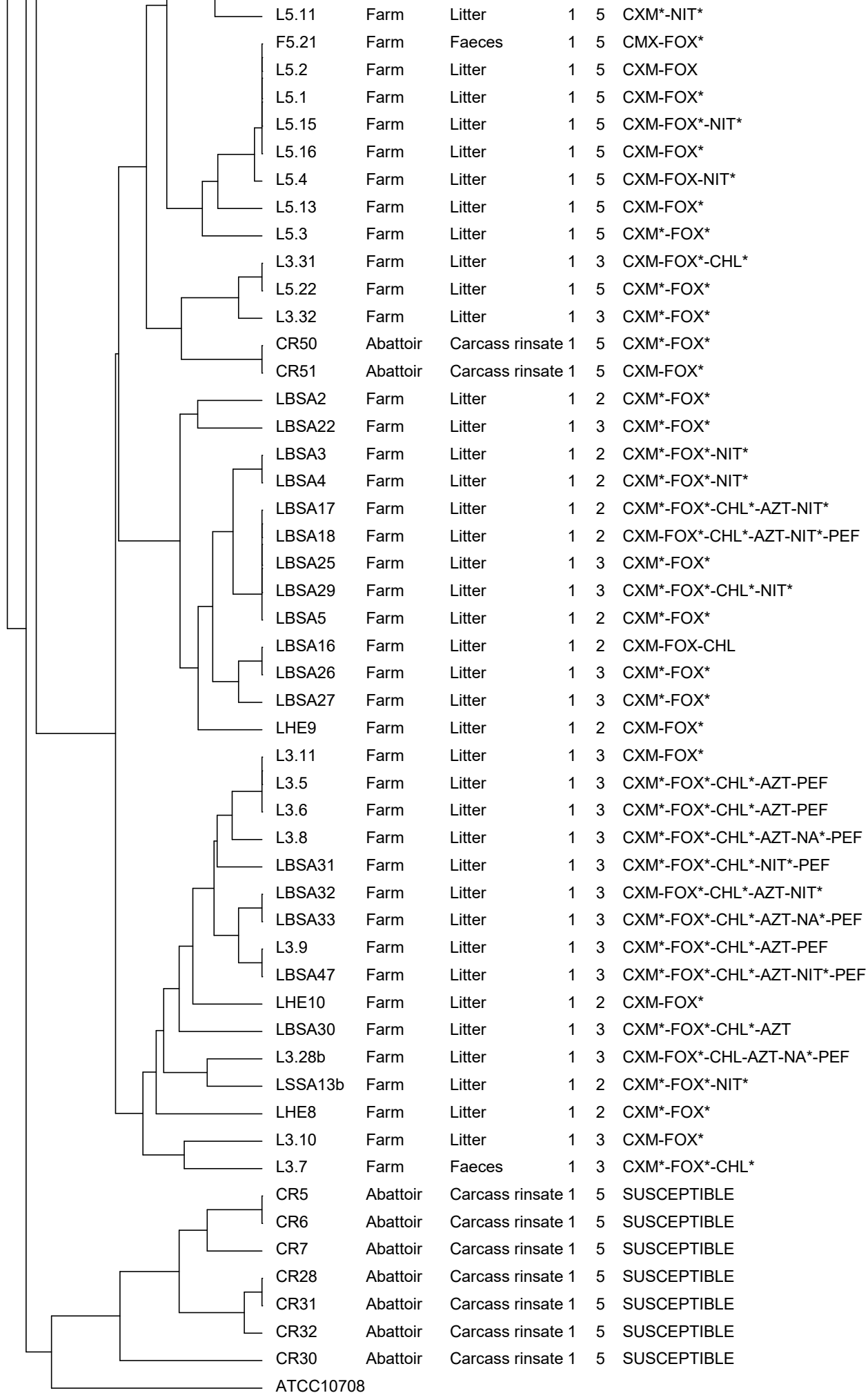

Supplement: Supplementary Materials — Table S1: source of Salmonella isolates. Figure S1: dendrogram of ERIC-PCR patterns constructed of Salmonella enterica isolates recovered from the farm-to-fork continuum. Salmonella enterica subsp. enterica serovar Choleraesuis ATCC 10708 was used as the quality control strain. . [file 5121273.f1.zip › Supplementary Figure S1.pdf]
